# *Toxoplasma* control of host apoptosis: the art of not biting too hard the hand that feeds you

**DOI:** 10.15698/mic2015.06.209

**Published:** 2015-05-30

**Authors:** Sébastien Besteiro

**Affiliations:** 1 DIMNP, UMR 5235 CNRS, Université de Montpellier, 34095 Montpellier, France.

**Keywords:** Toxoplasma, apoptosis, apoptosome, virulence, host control

*Toxoplasma gondii* is an obligate intracellular parasite that is able to infect a multitude of different vertebrate hosts and can survive in virtually any nucleated cell. The tachyzoite form of the parasite is very efficient at actively invading its host cells, where it establishes itself in a parasitophorous vacuole (PV). In this non fusogenic vacuole, the parasite is able to escape degradation from the endolysosomal system 1. The host endoplasmic reticulum and mitochondria are recruited to the periphery of the PV membrane shortly after invasion (Fig. 1), possibly as a source of nutrient for the parasite 2. Tachyzoites will subsequently undergo multiple rounds of cell division before being able to egress and, as a consequence, lyse the host cell (Fig. 1).

To develop a suitable replicating niche, *T. gondii* is able to modulate gene expression in its host cell 3. This is achieved through secretion of virulence factors from specialized organelles (Fig. 1): first, rhoptries are secreting so-called ROP proteins directly into the host cytosol as the parasite actively invades and forms the PV, then dense granules will release GRA proteins that will constitute a membranous nanotubular network inside the PV, or be exported at the PV membrane. These proteins are actively involved in the modulation of key host signaling pathways such as JAK/STAT (ROP16) 4, MAP kinase (ROP38, GRA24) 5,6, P53 (GRA16) 7 or NF-κB (GRA15) 8. The ability to interfere with these pathways has a profound impact on the immune and inflammatory responses from the host and thus on parasite survival and virulence. Indeed, most of the *Toxoplasma* strains found in Europe and North America are highly clonal, but they can be subdivided into three main lineages (I, II and III), which differ markedly for their pathogenicity *in vivo* and, because of a differential expression of the aforementioned virulence factors, they have different strategies to induce or disrupt the host immune response (see 9,10 for a review). Of course, the *in vivo* situation is very complex as it involves a variety of host tissue and cell types that provide a diverse, and more or less favorable, background for the parasite to dwell in.

**Figure 1:**
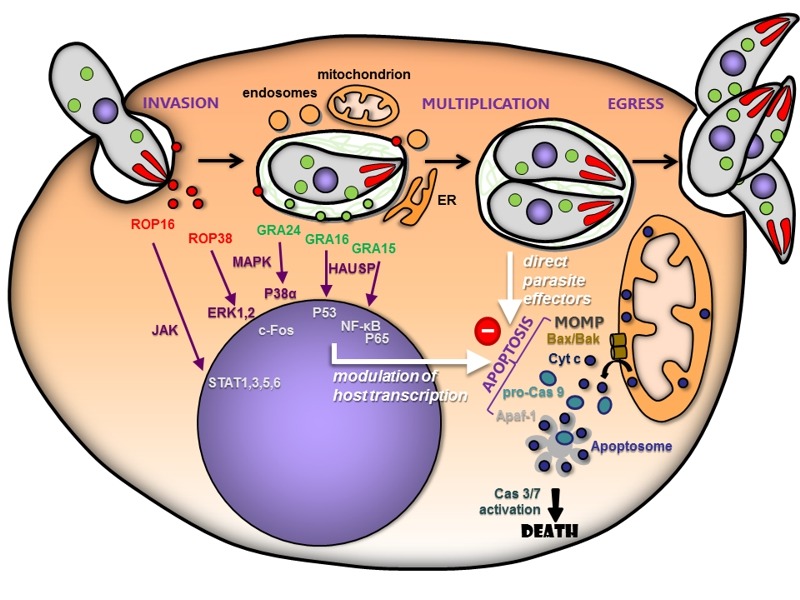
FIGURE 1: Schematic representation of *Toxoplasma* / host interactions, with a particular focus on the control of host apoptosis. The tachyzoite form actively invades its host cell and establishes itself in a parasitophorous vacuole in which it will multiply before egressing, and as a consequence destroy its host. Through the course of infection, the parasite is able to sequentially secrete rhoptry (ROP) and dense granule (GRA) proteins that will lead to the extensive modification of its host cell. Host organelles are recruited around the parasitophorous vacuole, potentially to serve as a nutrient source. The parasite is also able to modulate host protein expression by acting on host signaling pathways and transcription factors. This will influence host survival by altering the expression of key regulators of apoptosis, but as yet unidentified parasite effectors are also able to directly inhibit host apoptosis by interfering with apoptosome formation. MOMP: mitochondrial outer membrane permeabilization.

One important host pathway impacted by parasite factors is apoptosis. Apoptosis is a genetically-programmed cell death mechanism 11. There are two basic apoptotic signaling pathways: the extrinsic and the intrinsic pathways. The two apoptosis pathways converge on a common “execution” phase, which is driven by proteases known as caspases. Upon activation, these caspases cleave specific substrates and this will subsequently lead to the demise of the cell. The extrinsic pathway of apoptosis is initiated by the binding of death ligands such as the Fas ligand or TNF-α, to specific “death receptors” of the TNF receptor superfamily. The intrinsic pathway is activated by a variety of internal stimuli, including growth factor deprivation, DNA damage, oxidative stress, as well as invasion by pathogens. This pathway is largely centered around the mitochondria 12. Bcl-2 family members, such as Bax and Bak, trigger a mitochondrial outer membrane permeabilization (MOMP) event that will, in turn, lead to the release of mitochondrial intermembrane space proteins, including cytochrome *c* (Fig. 1). Once in the cytosol, cytochrome *c* binds an adaptor molecule called apoptotic protease-activating factor 1 (Apaf-1), leading to its oligomerization to form a heptameric structure called the apoptosome 13 (Fig. 1). The apoptosome recruits and activates pro-caspase-9 that, in turn, cleaves and activates the executioner caspases-3 and -7 in mammals.

As mentioned above, apoptosis is one of the ways mammalian cells can respond to an infection by pathogens 14. As the death of the infected cell is usually concomitant with the death of the infecting agent, self-destruction can promote efficient pathogen clearance. Several intracellular pathogens, including *T. gondii*, have thus apparently evolved means of interfering with the apoptosis machinery in order to keep their host alive, at least for the time needed for them to replicate efficiently. On the other hand, one should note that a highly virulent type I *Toxoplasma* strain is also known to be able to stimulate host apoptosis in splenic tissues 15, and one can imagine that inducing apoptosis specifically in lymphocytes and other immune cells can contribute to parasite immune evasion during the acute phase of infection. However, in permissive cell types, infection by *T. gondii* has been shown to render these cells generally resistant to multiple inducers of apoptosis 16. A number of subsequent studies have been suggesting that the parasite is manipulating the apoptosis machinery at multiple levels: inhibiting the release of cytochrome *c* from mitochondria to the host cell cytosol 17, affecting the balance of pro- and anti-apoptotic BCL-2 family members 18, directly interfering with caspase processing and function 19, blocking upstream signaling pathways 20, etc… The situation is quite complex and the mode of action is also probably depending on the parasite strains and host cell types that are being considered.

A recent report by Graumann *et al.* 21, gives new insights into the mechanisms of apoptosis inhibition by a type II *T. gondii* strain. Using a mitochondria-free *in vitro* reconstitution system, the authors demonstrate that there is a direct anti-apoptotic activity mediated by parasitic factors. More precisely, *Toxoplasma* protein lysates inhibit the binding of caspase 9 to Apaf-1, thereby abrogating caspase 9 activity and subsequent caspase 3/7 activation. Therefore, in addition to being able to inhibit cytochrome *c* release from host mitochondria, the parasite has another way of interfering with host apoptosis by directly preventing the recruitment of caspase 9 to the apoptosome.

Numerous factors, such as NF-κB or p53 22, have been reported to be involved in the regulation of apoptosis pathways and there are now increasing evidences that *Toxoplasma* can extensively modulate several of these transcription factors, especially for interfering with the host immune response. Although there is a clear impact of parasite infection on host cell apoptosis, the question remained if this was not after all, a mere consequence of the parasite trying to alter primarily other pathways of the host. For example, type II tachyzoites are known to be able to up-regulate the NF-κB pathway through the action of GRA15 8, which ultimately modulates the host immune response by the induction of cytokines such as interleukin 12, but this would also concomitantly lead to an inhibition of host apoptosis 20. The fact that parasite proteins are potentially able to specifically and directly interfere with the host apoptosis machinery, as shown by the work of Graumann *et al.* 21 and already suggested by other findings of the same group using parasite-secreted protein extracts 17, is a clue that there is an active manipulation of the pathway by *Toxoplasma*. It is a convincing evidence that the parasites would actively block host apoptosis to their advantage in order to keep their host cell alive during their own multiplication. Of course, the molecular identification of the parasitic factors interfering with apoptosome assembly is now eagerly anticipated, as well as knowing how they reach the host cell cytosol and can have access to the apoptosome machinery.

## References

[1] Jones TC, Hirsch JG (1972). The interaction between Toxoplasma gondii and mammalian cells. II. The absence of lysosomal fusion with phagocytic vacuoles containing living parasites.. J Exp Med.

[2] Sinai AP, Webster P, Joiner KA (1997). Association of host cell endoplasmic reticulum and mitochondria with the Toxoplasma gondii parasitophorous vacuole membrane: a high affinity interaction. J Cell Sci.

[3] Blader IJ, Koshy AA (2014). Toxoplasma gondii development of its replicative niche: in its host cell and beyond.. Eukaryot Cell.

[4] Saeij JPJ, Coller S, Boyle JP, Jerome ME, White MW, Boothroyd JC (2007). Toxoplasma co-opts host gene expression by injection of a polymorphic kinase homologue.. Nature.

[5] Peixoto L, Chen F, Harb OS, Davis PH, Beiting DP, Brownback CS, Ouloguem D, Roos DS (2010). Integrative genomic approaches highlight a family of parasite-specific kinases that regulate host responses.. Cell Host Microbe.

[6] Braun L, Brenier-Pinchart M-P, Yogavel M, Curt-Varesano A, Curt-Bertini R-L, Hussain T, Kieffer-Jaquinod S, Coute Y, Pelloux H, Tardieux I, Sharma A, Belrhali H, Bougdour A, Hakimi M-A (2013). A Toxoplasma dense granule protein, GRA24, modulates the early immune response to infection by promoting a direct and sustained host p38 MAPK activation.. J Exp Med.

[7] Bougdour A, Durandau E, Brenier-Pinchart M-P, Ortet P, Barakat M, Kieffer S, Curt-Varesano A, Curt-Bertini R-L, Bastien O, Coute Y, Pelloux H, Hakimi M-A (2013). Host cell subversion by Toxoplasma GRA16, an exported dense granule protein that targets the host cell nucleus and alters gene expression.. Cell Host Microbe.

[8] Rosowski EE, Lu D, Julien L, Rodda L, Gaiser RA, Jensen KDC, Saeij JPJ (2011). Strain-specific activation of the NF-kappaB pathway by GRA15, a novel Toxoplasma gondii dense granule protein.. J Exp Med.

[9] Melo MB, Jensen KDC, Saeij JPJ (2011). Toxoplasma gondii effectors are master regulators of the inflammatory response.. Trends Parasitol.

[10] Hunter CA, Sibley LD (2012). Modulation of innate immunity by Toxoplasma gondii virulence effectors.. Nat Rev Microbiol.

[11] Galluzzi L, Bravo-San Pedro JM, Vitale I, Aaronson SA, Abrams JM, Adam D, Alnemri ES, Altucci L, Andrews D, Annicchiarico-Petruzzelli M, Baehrecke EH, Bazan NG, Bertrand MJ, Bianchi K, Blagosklonny MV, Blomgren K, Borner C, Bredesen DE, Brenner C, Campanella M, Candi E, Cecconi F, Chan FK, Chandel NS, Cheng EH, Chipuk JE, Cidlowski JA, Ciechanover A, Dawson TM, Dawson VL, De Laurenzi V, De Maria R, Debatin K-M, Di Daniele N, Dixit VM, Dynlacht BD, El-Deiry WS, Fimia GM, Flavell RA, Fulda S, Garrido C, Gougeon M-L, Green DR, Gronemeyer H, Hajnoczky G, Hardwick JM, Hengartner MO, Ichijo H, Joseph B, Jost PJ, Kaufmann T, Kepp O, Klionsky DJ, Knight RA, Kumar S, Lemasters JJ, Levine B, Linkermann A, Lipton SA, Lockshin RA, López-Otín C, Lugli E, Madeo F, Malorni W, Marine J-C, Martin SJ, Martinou J-C, Medema JP, Meier P, Melino S, Mizushima N, Moll U, Muñoz-Pinedo C, Nuñez G, Oberst A, Panaretakis T, Penninger JM, Peter ME, Piacentini M, Pinton P, Prehn JH, Puthalakath H, Rabinovich GA, Ravichandran KS, Rizzuto R, Rodrigues CM, Rubinsztein DC, Rudel T, Shi Y, Simon H-U, Stockwell BR, Szabadkai G, Tait SW, Tang HL, Tavernarakis N, Tsujimoto Y, Vanden Berghe T, Vandenabeele P, Villunger A, Wagner EF, Walczak H, White E, Wood WG, Yuan J, Zakeri Z, Zhivotovsky B, Melino G, Kroemer G (2015). Essential versus accessory aspects of cell death: recommendations of the NCCD 2015.. Cell Death Differ.

[12] Gupta S, Kass GEN, Szegezdi E, Joseph B (2009). The mitochondrial death pathway: a promising therapeutic target in diseases.. J Cell Mol Med.

[13] Yuan S, Akey CW (2013). Apoptosome structure, assembly, and procaspase activation.. Struct Lond Engl 1993.

[14] Labbé K, Saleh M (2008). Cell death in the host response to infection.. Cell Death Differ.

[15] Gavrilescu LC, Denkers EY (2001). IFN-gamma overproduction and high level apoptosis are associated with high but not low virulence Toxoplasma gondii infection.. J Immunol Baltim Md 1950.

[16] Nash PB, Purner MB, Leon RP, Clarke P, Duke RC, Curiel TJ (1998). Toxoplasma gondii-infected cells are resistant to multiple inducers of apoptosis.. J Immunol Baltim Md 1950.

[17] Keller P, Schaumburg F, Fischer SF, Häcker G, Gross U, Lüder CGK (2006). Direct inhibition of cytochrome c-induced caspase activation in vitro by Toxoplasma gondii reveals novel mechanisms of interference with host cell apoptosis.. FEMS Microbiol Lett.

[18] Carmen JC, Hardi L, Sinai AP (2006). Toxoplasma gondii inhibits ultraviolet light-induced apoptosis through multiple interactions with the mitochondrion-dependent programmed cell death pathway.. Cell Microbiol.

[19] Vutova P, Wirth M, Hippe D, Gross U, Schulze-Osthoff K, Schmitz I, Lüder CGK (2007). Toxoplasma gondii inhibits Fas/CD95-triggered cell death by inducing aberrant processing and degradation of caspase 8.. Cell Microbiol.

[20] Molestina RE, Payne TM, Coppens I, Sinai AP (2003). Activation of NF-kappaB by Toxoplasma gondii correlates with increased expression of antiapoptotic genes and localization of phosphorylated IkappaB to the parasitophorous vacuole membrane.. J Cell Sci.

[21] Graumann K, Schaumburg F, Reubold T, Hippe D, Eschenburg S, Lüder C (2015). Toxoplasma gondii inhibits cytochrome c-induced caspase activation in its host cell by interference with holo-apoptosome assembly.. Microb Cell.

[22] Wong RS (2011). Apoptosis in cancer: from pathogenesis to treatment.. J Exp Clin Cancer Res.

